
JOURNAL INFORMATION
==============================
NLM Title Abbreviation: Curr Med Sci
Journal ID: Curr Med Sci
NLM Journal ID: 101729993

Current Medical Science

ISSN: 2096-5230
EISSN: 2523-899X

ARTICLE INFORMATION
==============================
PMCID: PMC7608338
PMID: 32337701
DOI: 10.1007/s11596-020-2187-z
Article ID: 2187
Article version: 2
Subjects: Erratum

Erratum to: Protein Phosphatase 2A as a Drug Target in the Treatment of Cancer and Alzheimer’s Disease

Wei Hui 510442763@qq.com
1
Zhang Hui-liang 734886938@qq.com
1
Xie Jia-zhao 1
Meng Dong-li 1
Wang Xiao-chuan 1
Ke Dan kedan@hust.edu.cn
1
Zeng Ji whzjmicro@163.com
2
Liu Rong 1
1 grid.33199.31 0000 0004 0368 7223 Department of Pathophysiology, Key Laboratory of Ministry of Education for Neurological Disorders, School of Basic Medicine, Tongji Medical College, Huazhong University of Science and Technology, Wuhan, 430030 China
2 grid.33199.31 0000 0004 0368 7223 Department of Clinic Laboratory, Wuhan Fourth Hospital, Tongji Medical College, Huazhong University of Science and Technology, Wuhan, 430030 China
Electronic publication date: 2020 Apr 26
Print publication date: 2020
Volume: 40
Issue: 2
First page: 389
Last page: 389
Copyright: © The Author(s) 2020
License: Open Access This article is distributed under the terms of the Creative Commons Attribution 4.0 International License (https://creativecommons.org/licenses/by/4.0/), which permits use, sharing, adaptation, distribution and reproduction in any medium or format, as long as you give appropriate credit to the original author(s) and the source, provide a link to the Creative Commons license, and indicate if changes were made.
License URL: https://creativecommons.org/licenses/by/4.0/

Erratum for: Protein Phosphatase 2A as a Drug Target in the Treatment of Cancer and Alzheimer's Disease. 40 1 13 3 2020 1 8 Curr Med Sci 10.1007/s11596-020-2140-1 32166659
issue-copyright-statement: © Huazhong University of Science and Technology 2020

==============================
The online version of the original article can be found at 10.1007/s11596-020-2140-1

The authors contributed equally to this work.

